# Methionine Antagonizes *para*-Aminosalicylic Acid Activity via Affecting Folate Precursor Biosynthesis in *Mycobacterium tuberculosis*

**DOI:** 10.3389/fcimb.2018.00399

**Published:** 2018-11-12

**Authors:** Michael D. Howe, Shannon L. Kordus, Malcolm S. Cole, Allison A. Bauman, Courtney C. Aldrich, Anthony D. Baughn, Yusuke Minato

**Affiliations:** ^1^Department of Microbiology and Immunology, University of Minnesota Medical School, Minneapolis, MN, United States; ^2^Department of Medicinal Chemistry, University of Minnesota, Minneapolis, MN, United States

**Keywords:** *Mycobacterium tuberculosis*, anti-folate drug, *para*-aminosalicylic acid, methionine, *para*-aminobenzoic acid, biotin, antagonism, methionine transport

## Abstract

*para*-Aminosalicylic acid (PAS) is a second-line anti-tubercular drug that is used for the treatment of drug-resistant tuberculosis (TB). PAS efficacy in the treatment of TB is limited by its lower potency against *Mycobacterium tuberculosis* relative to many other drugs in the TB treatment arsenal. It is known that intrinsic metabolites, such as, *para*-aminobenzoic acid (PABA) and methionine, antagonize PAS and structurally related anti-folate drugs. While the basis for PABA-mediated antagonism of anti-folates is understood, the mechanism for methionine-based antagonism remains undefined. In the present study, we used both targeted and untargeted approaches to identify factors associated with methionine-mediated antagonism of PAS activity. We found that synthesis of folate precursors as well as a putative amino acid transporter, designated MetM, play crucial roles in this process. Disruption of *metM* by transposon insertion resulted in a ≥30-fold decrease in uptake of methionine in *M. bovis* BCG, indicating that *metM* is the major facilitator of methionine transport. We also discovered that intracellular biotin confers intrinsic PAS resistance in a methionine-independent manner. Collectively, our results demonstrate that methionine-mediated antagonism of anti-folate drugs occurs through sustained production of folate precursors.

## Introduction

*Mycobacterium tuberculosis* is responsible for ~10.4 million new cases of active tuberculosis (TB) and 1.3 million deaths annually (World Health Organization, 2017). While TB chemotherapeutic intervention is highly successful in curing drug-susceptible TB infections, therapy is challenging, in part, because it requires a minimum of 6 months of treatment with drugs associated with adverse reactions. In addition, the emergence of drug-resistant strains of *M. tuberculosis* has dramatically increased the complexity and cost of TB treatment (Gandhi et al., 2006; Gehre et al., 2016). Therefore, the development of more efficacious TB chemotherapy regimens is imperative to improve treatment outcomes.

*para*-Aminosalicylic acid (PAS) was the second drug to be developed exclusively for TB chemotherapy (Lehmann, 1946). Although PAS was a cornerstone agent of early multidrug TB therapies, the introduction of more potent anti-tubercular agents into TB treatment regimens greatly diminished its usage (Minato et al., 2015). Emergence of *M. tuberculosis* strains with resistance to first-line anti-tubercular agents led to the resurgence of PAS as an second-line drug to treat infections that failed to respond to standard short-course therapy (Donald and Diacon, 2015). However, compared to many other anti-tubercular drugs, PAS is less potent and is associated with a high rate of gastrointestinal distress which limits its use to the treatment of multi-drug resistant TB for which there are few other treatment options (Zumla et al., 2013). Thus, it is important to develop novel strategies to enhance PAS potency, limit adverse reactions and improve treatment success rates.

**Figure 1:**
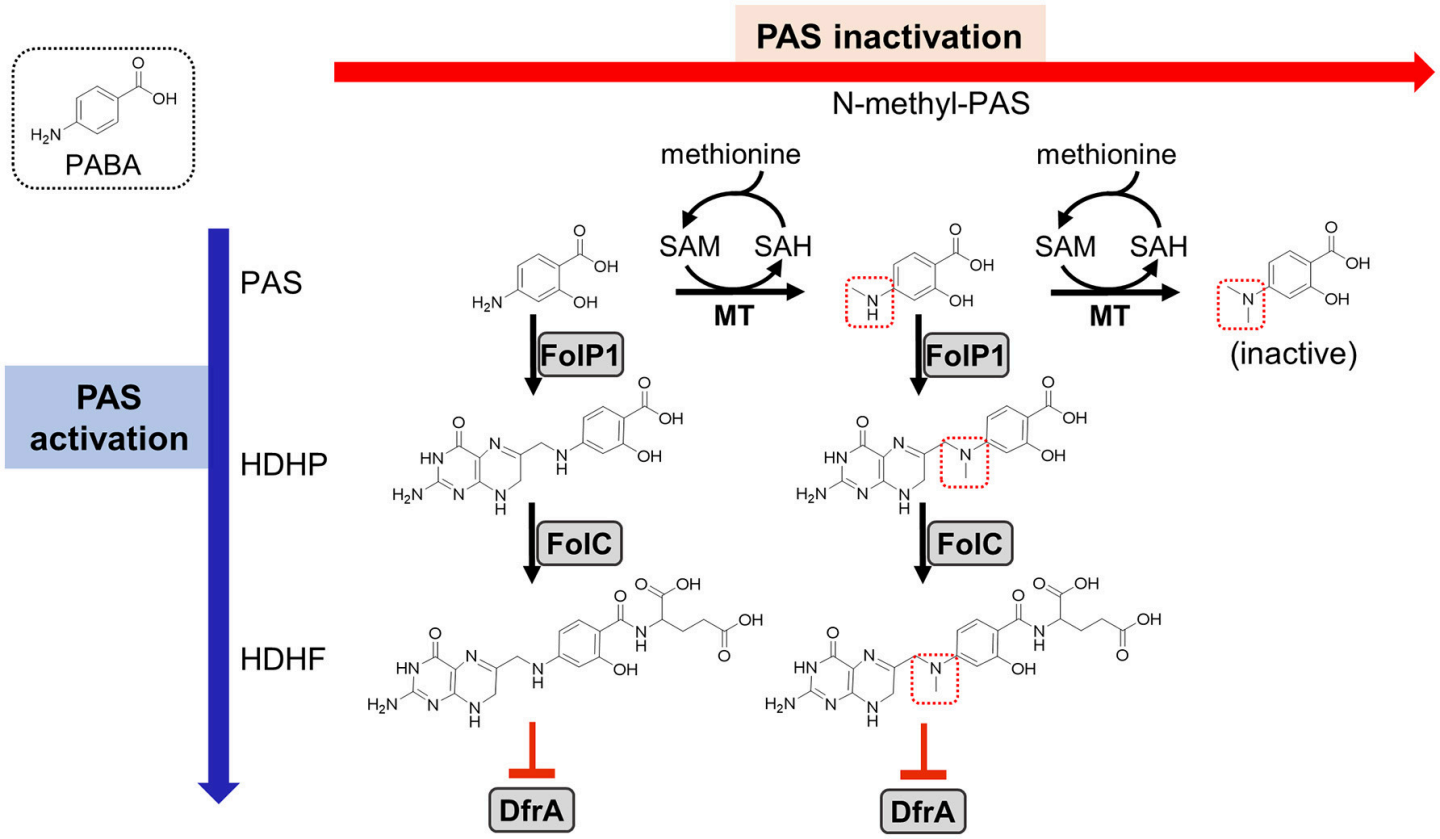
Previously proposed models for PAS activation and methionine-mediated PAS inactivation. As indicated on the left, PAS is an analog of PABA (shown in a dotted box) and is a prodrug that is activated by the *M. tuberculosis* folate biosynthetic pathway. PAS is incorporated *in lieu* of PABA by FolP1 and glutamylated by FolC to form the antimetabolite HDHF which inhibits DfrA activity (indicated as red blunted arrows) (Zheng et al., 2013; Zhao et al., 2014; Minato et al., 2015; Dawadi et al., 2017). Previous work has identified N-methyl and *N,N*-dimethyl PAS species (methyl groups indicated with dotted red boxes) in metabolite extracts from *M. tuberculosis* treated with PAS (Chakraborty et al., 2013). As *N,N*-dimethylation of PAS prevents incorporation by FolP1, the resulting metabolite is inactive (shown on the right). This activity is presumed to be dependent upon an as of yet unidentified SAM-dependent methyltransferase(s). Supplementation with methionine may increase SAM pools, which could be utilized by the methyltransferase(s) to inactivate PAS, thereby conferring resistance. PAS, *para*-aminosalicylic acid; PABA, *para*-aminobenzoic acid; HDHP, 2′-hydroxy-7,8-dihydropteroate; HDHF, 2′-hydroxy-7,8-dihydrofolate; FolP1, dihydropteroate synthase; FolC, dihydrofolate synthase; DfrA, dihydrofolate reductase; MT, methyltransferase; SAM, *S-*adenosyl methionine; SAH, *S*-adenosyl homocysteine.

Until recently, little was known regarding the mode of action of PAS. PAS is a selective antimetabolite of the *M. tuberculosis* folate metabolic pathway acting as a structural analog of the folate precursor *para*-aminobenzoic acid (PABA) (Chakraborty et al., 2013; Minato et al., 2015). PAS is sequentially converted to 2′-hydroxy-7,8-dihydropteroate and 2′-hydroxy-7,8-dihydrofolate by enzymes in the *M. tuberculosis* folate metabolic pathway (Figure 1). 2′-hydroxy-7,8-dihydrofolate has been shown to potently inhibit *M. tuberculosis* dihydrofolate reductase (DHFR), the final step in synthesis of tetrahydrofolate (Zheng et al., 2013; Zhao et al., 2014; Minato et al., 2015; Dawadi et al., 2017). Since PAS and PABA are comparable substrates for the folate biosynthetic pathway, supplementation of *M. tuberculosis* cultures with PABA antagonizes the inhibitory activity of PAS by outcompeting for ligation to 6-pyrophosphomethyl-7,8-dihydropterin (DHPPP) by dihydropteroate synthase (DHPS) (Youmans et al., 1947). We previously reported that intracellular PABA mediates intrinsic resistance to PAS in *M. tuberculosis*, and disruption of synthesis of this critical intermediate in folate biosynthesis can potentiate antifolate action, including that of sulfa drugs (Thiede et al., 2016).

Methionine is a potent antagonist of PAS in *M. tuberculosis* (Hedgecock, 1956), yet, the basis for this antagonism remains poorly understood. Because disruption of the folate pathway in *M. tuberculosis* results in depletion of metabolites within multiple essential folate-dependent pathways (Chakraborty et al., 2013; Nixon et al., 2014), supplementation with methionine alone is not expected to recover loss of folate pathway integrity. A recent study showed that PAS can be converted to *N*-methyl and *N,N*-dimethyl PAS species within *M. tuberculosis* cells (Figure 1; Chakraborty et al., 2013). *N*-methyl-PAS retains activity against *M. tuberculosis*, while *N*,*N*-dimethyl-PAS shows no anti-tubercular activity since the resulting tertiary amine is incapable of nucleophilically reacting with DHPPP during the first step of PAS bioactivation (Figure 1). Since addition of methionine can potentially enhance the ability of *M. tuberculosis* to methylate PAS by increasing *S*-adenosylmethionine (SAM) abundance, it is possible that methionine promotes inactivation of PAS through *N*,*N*-dimethylation by an unidentified methyltransferase.

In the present study we screened ~10,000 independent *Mycobacterium bovis* BCG transposon insertion mutants (BCG::*himar1*) to identify genetic determinants associated with methionine-mediated PAS antagonism. In parallel to analysis of BCG::*himar1* mutants, we characterized factors that affect PAS susceptibility in *M. tuberculosis* for their involvement in methionine-mediated PAS antagonism. Our findings reveal the importance of folate precursor biosynthesis and methionine transport in methionine-mediated PAS antagonism.

## Materials and methods

### Chemical reagents

All chemical reagents except for (S)-2-Amino-4-(methylthio-d_3_) butanoic Acid [L-methionine-(*methyl*-d_3_)] (Toronto Research Chemicals) and 2'-hydroxy-pteroate (pterin-PAS) were purchased from Sigma-Aldrich. Pterin-PAS was synthesized by Drs. Richard Lee and Ying Zhao at St Jude Children's Research Hospital by using a similar synthesis method reported elsewhere (Zhao et al., 2016).

### Bacterial strains and growth conditions

Bacterial strains utilized in this study are described in Table 1. Unless otherwise indicated, Mycobacterial strains were grown in Middlebrook 7H9 liquid medium supplemented with tyloxapol (0.05% vol/vol) or on Middlebrook 7H10 agar plates at 37°C. For *M. bovis* BCG and *M. tuberculosis* H37Ra, oleate-albumin-dextrose-catalase (OADC; Becton Dickinson 10% vol/vol), and glycerol (0.2% vol/vol) were supplemented to Middlebrook 7H9 and Middlebrook 7H10. For *Mycobacterium smegmatis* mc^2^155, Middlebrook 7H9, and Middlebrook 7H10 were amended with dextrose (0.2% vol/vol). *Escherichia coli* DH5α λ*pir* was grown in LB broth or on LB agar plate. When necessary, kanamycin or hygromycin were added to media at 50 and 150 μg/ml respectively for selection of mycobacterial and *E. coli* strains.

**Table 1:** List of bacterial strains used in this study.

**Strain**	**Relevant features**	**Reference or source**
*M. bovis* BCG Pasteur	Pasteur strain of spontaneously attenuated variant of *M. bovis*	Brosch et al., 2007
*M. bovis* BCG Pasteur *metM*::*himar1*	Transposon insertion mutant with disruption in *BCG_3282c* (*metM*)	This work
*M. bovis* BCG Pasteur *metM*::*himar1*/ pUMN002hyg::*metM*	Complemented *BCG_3282c* (*metM*) transposon insertion mutant	This work
*M. bovis* BCG Pasteur *bioB*::*himar1*	Transposon insertion mutant with disruption in *bioB*	This work
*M. tuberculosis* H37Ra	Spontaneously attenuated variant of *M. tuberculosis* strain H37Rv	Steenken et al., 1934
*M. tuberculosis* H37Ra Δ*pabB*	H37Ra strain with the *pabB* coding sequence replaced by a hygromycin resistance cassette	Thiede et al., 2016
*M. smegmatis* mc^2^155	Used for propagation of *himar1* mycobacteriophage	Snapper et al., 1990
*E. coli* DH5αλpir	Utilized to replicate self-ligated *himar1* plasmids for determination of transposon insertion site	Taylor et al., 1993

For sulfur utilization studies, a modified sulfate-free Sautons medium (Allen, 1998) was prepared with all inorganic sulfate salts (MgSO_4_ and ZnSO_4_) replaced with inorganic chloride salts (MgCl and ZnCl) keeping the concentrations of Mg^2+^ and Zn^2+^ ions the same. For the characterizations of the biotin auxotroph mutant, biotin-free 7H9 medium was prepared. The biotin-auxotrophic strain, *M. bovis* BCG *bioB*::*himar1*, was maintained in the biotin-free 7H9 medium supplemented with 0.5 μg/ml biotin. The PABA-auxotrophic strain H37Ra Δ*pabB* was maintained in 7H9 medium supplemented with PABA (10 ng/ml). For experiments involving PABA limitation, PABA-free 7H9 medium was prepared in glassware that was baked at 300°C for 1 h to remove residual PABA before use.

### *M. bovis* BCG::*himar1* mutant library construction and screening

The phAE180 mycobacteriophage containing a *mariner* transposable element, *himar1*, with a kanamycin resistance cassette was used to transduce *M. bovis* BCG creating a library of BCG::*himar1* mutants as described previously (Rubin et al., 1999; Kriakov et al., 2003). Transduced cells were plated onto 7H10 agar containing kanamycin and 10 μg/ml methionine. ~10,000 mutant strains were screened by picking and patching onto 7H10 agar supplemented with methionine (Met plates) and onto 7H10 agar plates additionally amended with 5 μg/ml PAS (Met-PAS plates). Mutant strains that grew on the Met plates, but were inhibited for growth the Met-PAS plates, were selected for secondary screening following the same protocol. PAS susceptibility was assessed for strains that passed the secondary screen. *himar1* insertion sites were determined as previously described (Rubin et al., 1999). Briefly, extracted genomic DNA was digested with *Bss*HII and self-ligated to produce circular DNAs. The circularized DNAs that contained *ori6K* from a part of *himar1* transposon were used to transform *E. coli* DH5αλpir. Plasmids were purified from the transformants. Sequences of genomic DNA adjacent to the 3′ end of the *himar1* transposon insertion site were determined by Sanger sequencing (performed by Eurofins) using the KanSeq_Rev (5′-GCATCGCCTTCTATCGCCTTC-3′) primer (Baughn et al., 2010). Insertion site locations were determined by aligning the resulting sequence files with the *M. bovis* BCG Pasteur genome sequence (GenBank accession number NC_008796).

### Construction of *metM* complemented strain

Complementation of the *metM*::*himar1* strain was accomplished by transforming the mutant with a replicative mycobacterial expression vector constitutively expressing *metM* under the control of the P_smyc_ promoter (Ehrt et al., 2005). Since the *himar1* transposable element encodes a kanamycin resistance gene, a hygromycin resistant version of the replicative mycobacterial expression vector pUMN002 was constructed (Peterson et al., 2015). The hygromycin resistance cassette was amplified from the plasmid p0004S by PCR with primers p0004s_Hygro+P_F and p0004s_Hygro+P_R (Supplementary Table S1). The entire plasmid except for the kanamycin resistance cassette of pUMN002 was amplified by PCR using primers pTIC6a_F and pTIC6a_R. The linear vector and hygromycin cassette were digested with either *Sbf* I or *Afl*II and ligated to produce pUMN002hyg. The *metM* coding region (*BCG_3282c*) was amplified from *M. bovis* BCG genomic DNA by PCR with primers BCG3282c*_*For and BCG3282c_Rev (Supplementary Table S1), digested with *Nhe*I and *Eco*RI, and ligated into pUMN002hyg. The *E. coli* transformants of the resultant plasmid pUMN002hyg::*metM* were selected on LB plates containing hygromycin. After verification of the plasmid, the *M. bovis* BCG *metM*::*himar1* disruption strain was transformed with the pUMN002hyg::*metM* by electroporation and selected for on supplemented 7H10 agar plates containing hygromycin and kanamycin.

### Determination of minimum inhibitory concentrations

The minimum inhibitory concentrations (MIC) of anti-tubercular compounds were determined as previously described (Dillon et al., 2014). Briefly, for determination of the MIC in liquid culture, 2-fold dilution series of drugs in 7H9 medium were prepared. Logarithmically growing Mycobacterium strains were inoculated into the drug-containing 7H9 medium in 30-ml square bottles (Nalgene) to an optical density (OD_600_) of 0.01. OD_600_ were measured after shaking (100 rpm) at 37°C for 14 days. The liquid MIC_90_ was defined as the minimum concentration of drug required to inhibit at least 90% of growth relative to growth in the no-drug control cultures. For determination of the agar plate MIC, logarithmically growing *M. bovis* BCG strains were serially-diluted and inoculated onto 7H10 agar plates containing drug in 2-fold dilution series. The agar plate MIC was determined by visually inspecting growth relative to growth on the no-drug control plates after grown at 37°C for 21 days. All anti-tubercular compounds employed in this study were dissolved in DMSO. The highest concentration of DMSO in the growth media was 2.5%.

### Analysis of growth kinetics

Logarithmically growing Mycobacterium strains were washed twice in an equal volume of fresh medium. Cells were diluted to an OD_600_ of 0.01 in 30-ml square bottles (Nalgene) and supplements with or without drug were added at the described concentrations. Cultures were shaken (100 rpm) and OD_600_ were measured at various time points over a 14-day time-course.

### Methionine utilization assays

*M. bovis* BCG strains were grown to mid-log phase in 7H9 broth and washed twice with sulfate-free Sautons medium. Resuspended cells were diluted to an OD_600_ of 0.01 in sulfate-free Sautons medium. Cultures were then incubated for 5 days to exhaust remaining sulfur. Exhausted cells were aliquoted into 30-ml square bottles (Nalgene) and sulfur-containing metabolites were added at the given concentrations. Cultures were incubated at 37°C and shaken (100 rpm). The fold-change in OD_600_ (as a ratio of the final OD_600_/initial OD_600_) was assessed following 1 week of incubation after the addition of metabolites.

### Methionine uptake assays

*M. bovis* BCG strains were grown until mid-log phase (OD_600_ 0.4–0.6). Cells were then diluted to OD_600_ 0.3 and divided into three 40 ml assay mixtures for each condition tested. Each assay mixture was incubated at 37°C for 2 h with shaking (100 rpm) in the presence or absence of L-methionine-(*methyl*-d_3_) and/or carbonylcyanide m-chlorophenylhydrazone (CCCP). L-methionine-(*methyl*-d_3_) and CCCP were added at 50 μM and 100 μM, respectively. Following 2 h of incubation, each assay mixture culture was pelleted by centrifugation at 3000 *g* for 10 min and then washed three times with ice-cold phosphate buffered saline (pH 7.4). Metabolites were extracted by using 1 ml of acetonitrile:methanol:water (40:40:20) extraction buffer as previously described (Chakraborty et al., 2013). Following extraction, 350 μL of each sample was concentrated *in vacuo* and reconstituted in 90 μL of 10 mM ammonium formate (80:20 MeCN:H_2_O) containing 1 μM internal standard (N-phenyl glycine). Quantitation was performed using a Shimadzu UFLC-XR system and an AB Sciex QTRAP 5500 mass spectrometer. L-methionine-(*methyl*-d_3_) (Toronto Research Chemicals) was used as an authentic standard. N-phenyl glycine (Chem-Impex) was used as an internal standard. LC was performed using a SeQuant ZIC-pHILIC column (4.6 × 150 mm, 5 μm particle size; Millipore Sigma). Mobile phase A was 10 mM ammonium formate in 20:80 MeCN:H_2_O, and mobile phase B was 10 mM ammonium formate in 80:20 MeCN:H_2_O. The LC method was as follows: 100% B from 0.0 to 0.10 min, then decreasing from 100% B to 82.5% B from 0.10 to 22.00 min, followed by a decrease from 82.5% B to 0% B from 22.00 to 23.00 min. 0% B was maintained from 23.00 to 24.00 min, then increased to 100% B from 24.00 to 25.00 min. The column was then re-equillibrated at 100% B from 25.00 to 30.00 min, for a total run time of 30 min. The flow rate was 600 μL/min, the injection volume was 10 μL, and the column oven was maintained at 35°C. Analytes were monitored by positive mode MS via multiple reaction monitoring (MRM). MS settings were optimized via infusion of analytes in 10 mM ammonium formate (80:20 MeCN:H_2_O); L-methionine-(*methyl*-d_3_) was infused at 5 μM and 2 μL/min flow rate, and N-phenyl glycine was infused at 1 μM and 2 μL/min. Mass transitions monitored and MS conditions are summarized in Supplementary Table S2. Analyte and internal standard peak areas were quantitated using MultiQuant software (Version 2.0.2); analyte peak areas were normalized to internal standard peak areas. A standard curve was prepared for methionine-(*methyl*-d_3_) with 2-fold serial dilutions from 25.00 to 0.39 μM and used to determine concentrations of methionine-(*methyl*-d_3_) in samples. Zero concentration standards were also prepared and used to determine the lower limits of detection and quantitation, which were defined as 3:1 and 10:1 signal-to-noise ratios, respectively. Concentrations of methionine-(*methyl*-d_3_) were normalized to the protein concentration of each sample as determined by using the BCA protein assay kit (Pierce Biotechnology).

## Results

### Identification of *M. bovis* BCG genes involved in methionine-mediated antagonism of PAS

**Figure 2:**
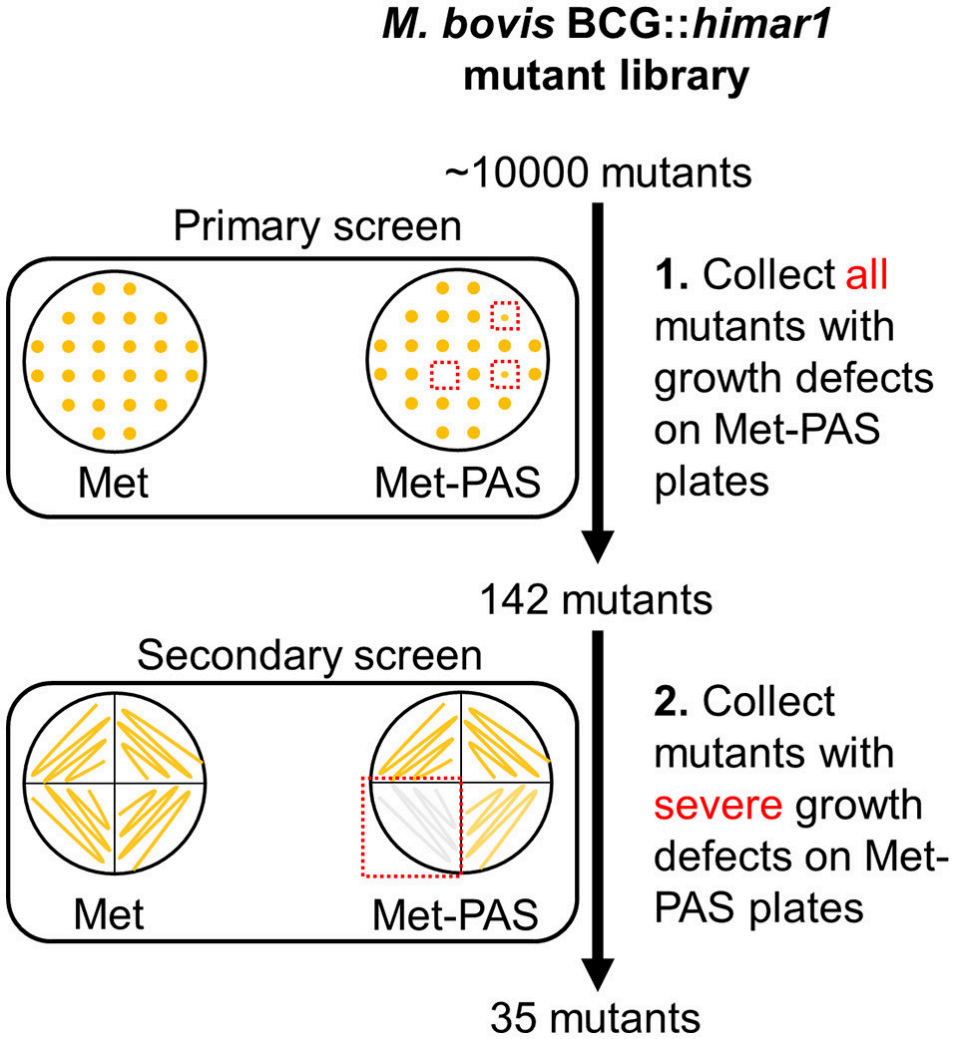
Schematic representation of genome-wide transposon mutagenesis of *M. bovis* BCG and Met-PAS screening method. *M. bovis* BCG::*himar1* mutants (~10,000 mutants) were patched onto Met and Met-PAS plates. Clones with observable growth defects on Met-PAS plates were subjected to secondary screening. These 35 mutants that passed the secondary screening were collected and insertion site locations were determined.

A library of *M. bovis* BCG transposon insertion mutant strains was constructed using the phAE180 mycobacteriophage containing a *mariner*-family transposable element. To identify genes associated with methionine-mediated PAS antagonism, ~10,000 BCG::*himar1* mutants were screened following the approach outlined in Figure 2. Determination of the PAS MIC on 7H10 agar plates confirmed that 0.25 μg/ml was sufficient to fully inhibit growth of the *M. bovis* BCG parental strain. Screening was then undertaken on 7H10 agar plates containing 10 μg/ml methionine and 5 μg/ml PAS (Met-PAS plate). Growth of *M. bovis* BCG on Met-PAS plates was identical to growth seen on control 7H10 plates, which confirmed methionine-mediated PAS antagonism.

BCG::*himar1* insertion mutants which exhibited observable growth inhibition on the Met-PAS plates in comparison to the growth on 7H10 agar plates containing 10 μg/ml methionine (Met plate) were isolated. We then identified the *himar1* insertion sites within the 35 BCG::*himar1* mutants that had reproducible growth defects on Met-PAS plates compared to the growth on Met plates (Figure 2 and Table 2). Among these mutants, one strain with a *himar1* insertion located within *BCG_3282c*, encoding a putative amino acid/polyamine/organocation (APC) superfamily transporter of the CAT (cationic amino acid transporter) family (TC 2.A.3.3) (Jack et al., 2000; Elbourne et al., 2017), showed the most severe growth defect on Met-PAS plates suggesting BCG_3282c plays a major role in methionine-mediated antagonism of PAS. We also assessed the susceptibility of each mutant strain to PAS by measuring PAS MICs on 7H10 agar plates (Table 2). We observed the *BCG_3282c* mutant possessed wild-type PAS susceptibility suggesting this mutation is associated exclusively with methionine-mediated PAS antagonism.

**Table 2:** Sequence-validated gene insertions that affect PAS susceptibility in the presence or absence of antagonistic concentrations of methionine.

**Disrupted gene (*M. tuberculosis* H37Rv homolog)**	**Predicted function**	**Increased PAS susceptibility*^a^***	**Growth on PAS-Met*^b^***
*M. bovis* BCG	Wild-type	no	++++
*BCG_3282c* (*Rv3253c*)	Amino acid permease	no	–
*ftsH* (*ftsH*)	Membrane-bound protease (insertion located near upstream folate biosynthesis operon)	yes	+
*bioB* (*bioB*)	Biotin synthase involved in biotin biosynthesis	yes	+
*metB* (*metB*)	Cystathionine gamma-synthase involved in methionine biosynthesis	yes	+
*BCG_1906c* (*Rv1870c*)	Unknown	yes	+
*cysQ* (*cysQ*)	Sulfate assimilation pathway regulator	no	+
*accD2* (*accD2*)	Acetyl-CoA carboxylase involved in mycolic acid biosynthesis	no	+
*BCG_1988c-1989c* (*Rv1949c-Rv1950c*)	Unknown (conserved hypotheticals) (insertion is located within the intergenic region)	no	+
*PPE11*(*PPE11*)	Unknown (PPE family protein)	no	+
*arsC* (*arsC*)	Protein involved in arsenate resistance	no	+
*mmpL7* (*mmpL7*)	Phthiocerol dimycocerosate transporter	no	+
*BCG_2043c* (*Rv2024c*)	Unknown	no	+
*BCG_3116* (*Rv3091*)	Unknown	no	+
*BCG_0914c* (*Rv0862c*)	Unknown	no	+
*papA2* (*papA2*)	Protein involved in sulfolipid-1 biosynthesis	no	+
*BCG_2017* (*Rv2000*)	Unknown	no	+
*BCG_1401* (*Rv1339*)	Unknown	no	+
*BCG_1635* (*Rv1597*)	Unknown	no	+
*BCG_1082* (*Rv1026*)	Protein involved in polyphosphate regulation	no	+
*BCG_2497c* (*Rv2477c*)	Macrolide exporter	no	++
*BCG_3185c* (*Rv3161c*)	Dioxygenase	no	++
*BCG_0233* (*Rv0196*)	Transcriptional regulator	no	++
*BCG_3873* (*Rv3811*)	Cell surface protein involved in virulence	no	++
*BCG_1897* (*Rv1861*)	Conserved transmembrane protein	no	++
*BCG_2026* (*vapB15*)	Antitoxin component of an toxin-antitoxin operon with BCG_2027 (*vapC15*)	no	++
*kgtP* (*kgtP*)	Ketoglutarate transport protein	no	++
*mbtJ* (*mbtJ*)	Protein involved in mycobactin biosynthesis	no	++
*BCG_1492* (*Rv1431*)	Conserved membrane protein	no	++
*thiG* (*thiG*)	Protein involved in thiamine biosynthesis	no	++
*BCG_3826c* (*3767c*)	SAM-dependent methyltransferase which may be involved in Polyketide synthesis	no	++
*BCG_0424* (*Rv0386*)	Transcriptional regulator	no	++
*upp-sapM* (*upp-sapM*)	Proteins involved in pyrimidine the salvage pathway and arresting phagosomal maturation, respectively (insertion is located within the intergenic region of these two genes)	no	++
*fadD2* (*fadD2*)	Fatty-acid CoA Ligase	no	++
*PPE33a* (*PPE33a*)	Unknown (PPE family protein)	no	++
*esxJ* (*esxJ*)	Unknown	no	++

a *PAS susceptibility was assessed by determining the minimum concentration (MIC) of drug required to inhibit growth on 7H10 agar plates. The M. bovis BCG PAS MIC was found to be 0.25 μg/ml*.

b *Growth of M. bovis BCG transposants on methionine (10 μg/ml) only plates compared visually to plates containing methionine (10 μg/ml) and PAS (5 μg/ml) to screen for transposon insertion mutants susceptible to PAS-methionine treatment. (++++) represents no growth difference between PAS-methionine and methionine only plates (WT BCG). (++) represents ~50% impairment in growth. (+) represents 25% or less growth. (–) represents no growth observed*.

Although most mutant strains that were analyzed showed a similar level of PAS tolerance as the parent *M. bovis* BCG, four mutants (with transposon insertions in *bioB, ftsH, metB*, and *BCG_1906c*) were found to be more susceptible to PAS in the absence of methionine, indicating that the disrupted genes may be involved in intrinsic resistance to PAS (Table 2).

### MetM is essential for methionine-mediated antagonism of PAS

Based upon the observation that methionine failed to antagonize PAS activity in the *BCG_3282c* mutant, we hypothesized that BCG_3282c is the primary methionine transporter. We designated *BCG_3282c* herein as *metM* (methionine transporter for Mycobacterium) and further characterized the function of this gene. The *himar1* insertion was located near the 5′ end of the coding region for *metM* resulting in a 401 residue truncation of the 495 residue coding sequence, suggesting functional gene disruption by *himar1* insertion. Similar to the majority of transporters within the APC superfamily, MetM is predicted to possess 12 transmembrane spanning α-helices (Elbourne et al., 2017). *metM* is also highly conserved in the Mycobacterium genus, sharing 100% sequence identity with numerous *M. tuberculosis* complex organisms including *Rv3253c*, an ortholog from the virulent reference strain H37Rv. However, no close orthologs of *metM* have been structurally or functionally characterized thus far.

**Figure 3:**
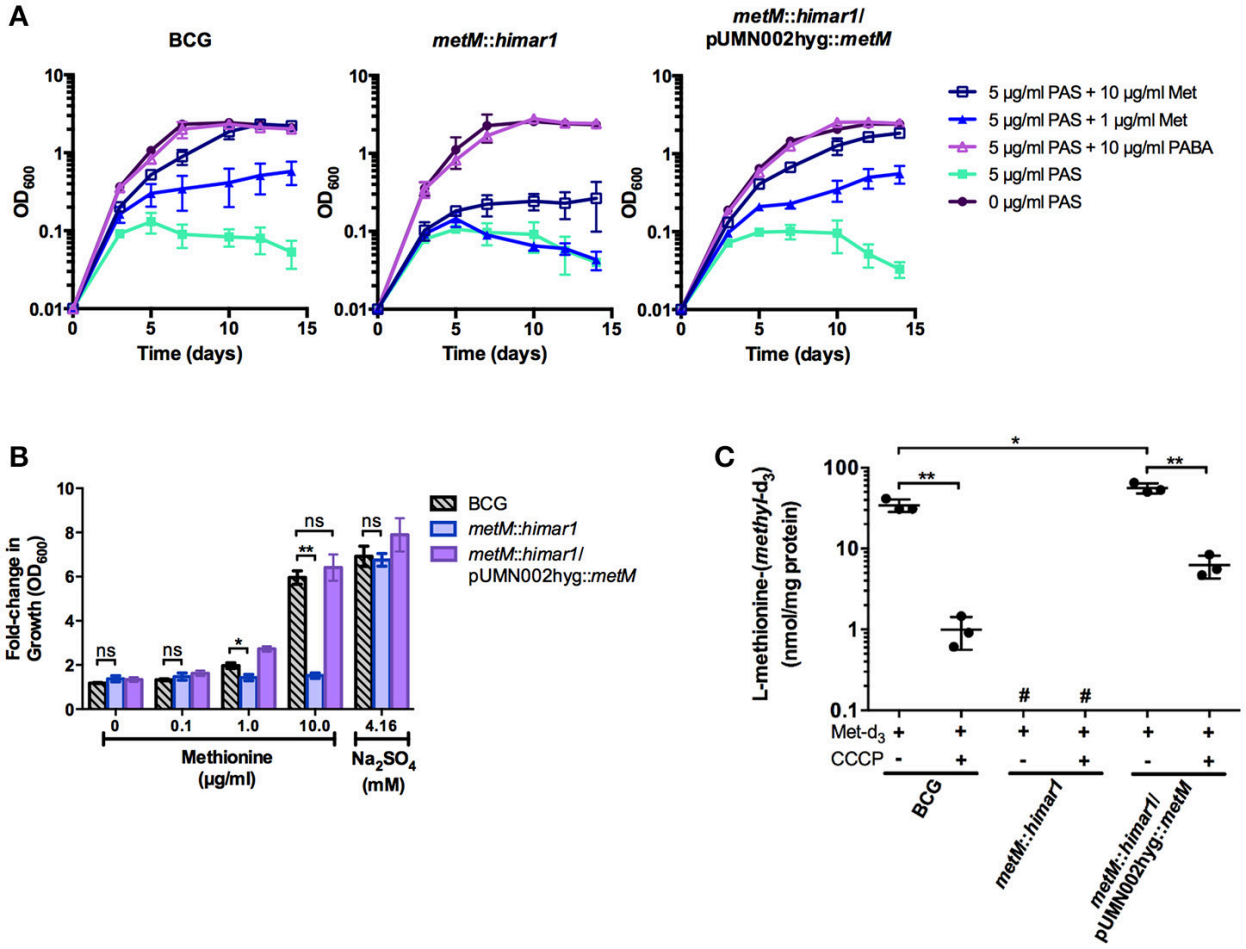
MetM is essential for methionine-mediated PAS antagonism and uptake of methionine in *M. bovis* BCG. **(A)** Growth kinetics of *M. bovis* BCG *metM*::*himar1* and *M. bovis* BCG wild type during PAS exposure when antagonistic metabolites are added. Growth was assessed by OD_600_ readings every 2–3 days. **(B)** *M. bovis* BCG strains were grown to an OD_600_ of ~0.5, washed three times to remove residual sulfate with sulfate-free Sautons medium, and resuspended in sulfate-free Sautons medium to a starting OD_600_ of 0.01 and cells were starved for sulfur for 5 days. Following the exhaust period, sulfur-sources were added, and cells were incubated for 7 days to resume growth. The fold-change in growth was assessed as a ratio of the final OD_600_ over the starting OD_600_ following the exhaust period (final OD_600_/starting OD_600_). **(C)** Uptake of L-methionine-(*methyl*-d_3_) was measured using LC-MS/MS. Samples that fell outside the quantifiable lower limit of the standard curve for L-methionine-(*methyl*-d_3_) (25.00–0.39 μM) were labeled #, not quantifiable. **(B,C)** *p*-values of pairwise comparisons (denoted by bracketed lines) were calculated using the Student *t*-test. ^*^p < 0.05, ^**^p < 0.005, ns indicates no significant difference (*p* > 0.05). Error bars denote standard deviation and are representative of 3 separate experiments.

To confirm whether *metM* disruption altered methionine antagonism or susceptibility to antimycobacterial drugs, susceptibility testing was conducted in liquid medium (Figure 3A and Supplementary Table S3). Growth of both wild type *M. bovis* BCG and the *metM*::*himar1* strain was fully inhibited by 5 μg/ml PAS. Addition of methionine restored growth during PAS treatment of wild-type *M. bovis* BCG in a dose-dependent manner. In contrast, growth of the *metM*::*himar1* strain was still inhibited by PAS even in the presence of 10 μg/ml methionine. When the *metM*::*himar1* strain was transformed with a plasmid expressing *metM*, addition of methionine growth in the presence of PAS, similar to the wild type strain indicating that MetM plays a role in methionine mediated PAS antagonism (Figure 3A). We were also able to eliminate the possibility that the *metM*::*himar1* strain was auxotrophic for methionine as both strains were able to grow in standard 7H9, a methionine-free medium. These strains were equally inhibited by the first-line antitubercular drugs rifampicin and isoniazid (Supplementary Table S3). PABA, another PAS antagonist, reversed PAS-mediated growth inhibition in both the wild type *M. bovis* BCG and the *metM*::*himar1* strain, validating that the *metM*::*himar1* strain is specifically impaired for methionine-mediated PAS antagonism.

### MetM is a major methionine transporter in *M. bovis* BCG

*M. tuberculosis* is known to utilize reverse transsulfuration to assimilate sulfur from methionine which can serve as the sole source of sulfur for this bacterium (Wheeler et al., 2005). Therefore, we tested whether disruption of *metM* would affect the ability of the bacilli to assimilate sulfur derived from methionine. When *M. bovis* BCG, the *metM*::*himar1* mutant strain and the *metM* complemented *metM*::*himar1* mutant strain were grown in sulfate-free Sautons medium, growth of all strains was limited (maximum OD_600_ = 0.3). Upon addition of sodium sulfate to the medium, strains resumed growth and achieved typical growth yields confirming these strains were previously starved for sulfur (Figure 3B). When methionine was added to sulfur starved *M. bovis* BCG, growth also resumed in a dose-dependent manner producing similar growth yields as compared to the addition of sulfate alone. In contrast, growth of the *metM*::*himar1* disruption strain could not be restored in the presence of methionine as the sole source of sulfur, but could be fully restored by *metM* complementation, indicating that MetM is required for methionine utilization.

To confirm our hypothesis that MetM is the primary methionine transporter in *M. bovis* BCG, we developed a methionine uptake assay using L-methionine-(*methyl*-d_3_) as the transport substrate. After *M. bovis* BCG strains were incubated with L-methionine-(*methyl*-d_3_), metabolites were extracted and then analyzed by LC-MS/MS to determine intracellular accumulation of L-methionine-(*methyl*-d_3_). We found that wild-type *M. bovis* BCG showed substantial methionine-(*methyl*-d_3_) accumulation compared to the *metM*::*himar1* mutant, (~50–100 fold decrease) whose samples fell below the lower limit of quantitation (Figure 3C). Complementation of the transporter significantly increased accumulation of methionine-(*methyl*-d_3_) (2-fold increase) over the same period of as wild-type BCG. Addition of the membrane uncoupler, CCCP, reduced methionine-(*methyl*-d_3_) uptake in all strains to a significant degree suggesting that the driving force for L-methionine transport is membrane potential. For all strains tested, control samples without methionine-(*methyl*-d_3_) added were found to lack detectable quantities of methionine-(*methyl*-d_3_) (data not shown). These findings indicate that MetM is an energy-dependent methionine transporter that plays a major role in methionine transport in *M. bovis* BCG.

### PABA biosynthesis is indispensable for methionine-mediated PAS antagonism in *M. tuberculosis*

Our large-scale screening failed to identify genes directly involved in methionine-mediated PAS antagonism. Thus, it is possible that genes involved in this process are redundant or are essential for *M. bovis* BCG under the growth conditions that were employed. Because addition of methionine can increase SAM levels and many *M. tuberculosis* SAM-dependent methyltransferase genes are essential, we investigated whether the ability to methylate PAS plays a role in methionine-mediated PAS antagonism. To test this, we evaluated whether methionine can antagonize the activated PAS species, 2′-hydroxy-pteroate (pterin-PAS), in *M. bovis* BCG. It is known that *N*,*N*-dimethyl-PAS has no anti-tubercular activity, presumably because *N*,*N*-dimethyl-PAS cannot react with DHPPP during the first step of PAS bioactivation (Figure 1). Thus, once PAS is activated to pterin-PAS, *N-*methylation should not affect its anti-tubercular activity. We confirmed pterin-PAS was active against wild-type *M. bovis* BCG at a comparable molar concentration to PAS (Table 3). Surprisingly, pterin-PAS was still potently antagonized by methionine suggesting that methionine-mediated PAS antagonism does not occur by *N*-methylation of PAS.

**Table 3:** Antagonism of PAS and pterin-PAS by methionine.

	**PAS MIC**_**90**_***^a^***		**Pterin-PAS MIC**_**90**_	
**Strain**	**–Met**	**+Met*^b^***	**–Met**	**+Met**
*M. bovis BCG*	1 (6.53)	>250 (1630)	5 (15.1)	>20 (61.5)
*M. tuberculosis* H37Ra	0.6 (3.92)	>250 (1630)	ND	ND
*M. tuberculosis* H37Ra Δ*pabB*	0.15 (0.98)	0.3 (1.96)	ND	ND

a *MIC_90_ is defined as the minimum concentration of drug required to restrict at least 90% of growth relative to growth seen in the no-drug control cultures. MIC90 are shown in μg/ml (μM)*.

*ND, not determined; Met, methionine; PAS, para-aminosalicylic acid*.

b *Methionine was supplemented at 10 μg/ml*.

**Figure 4:**
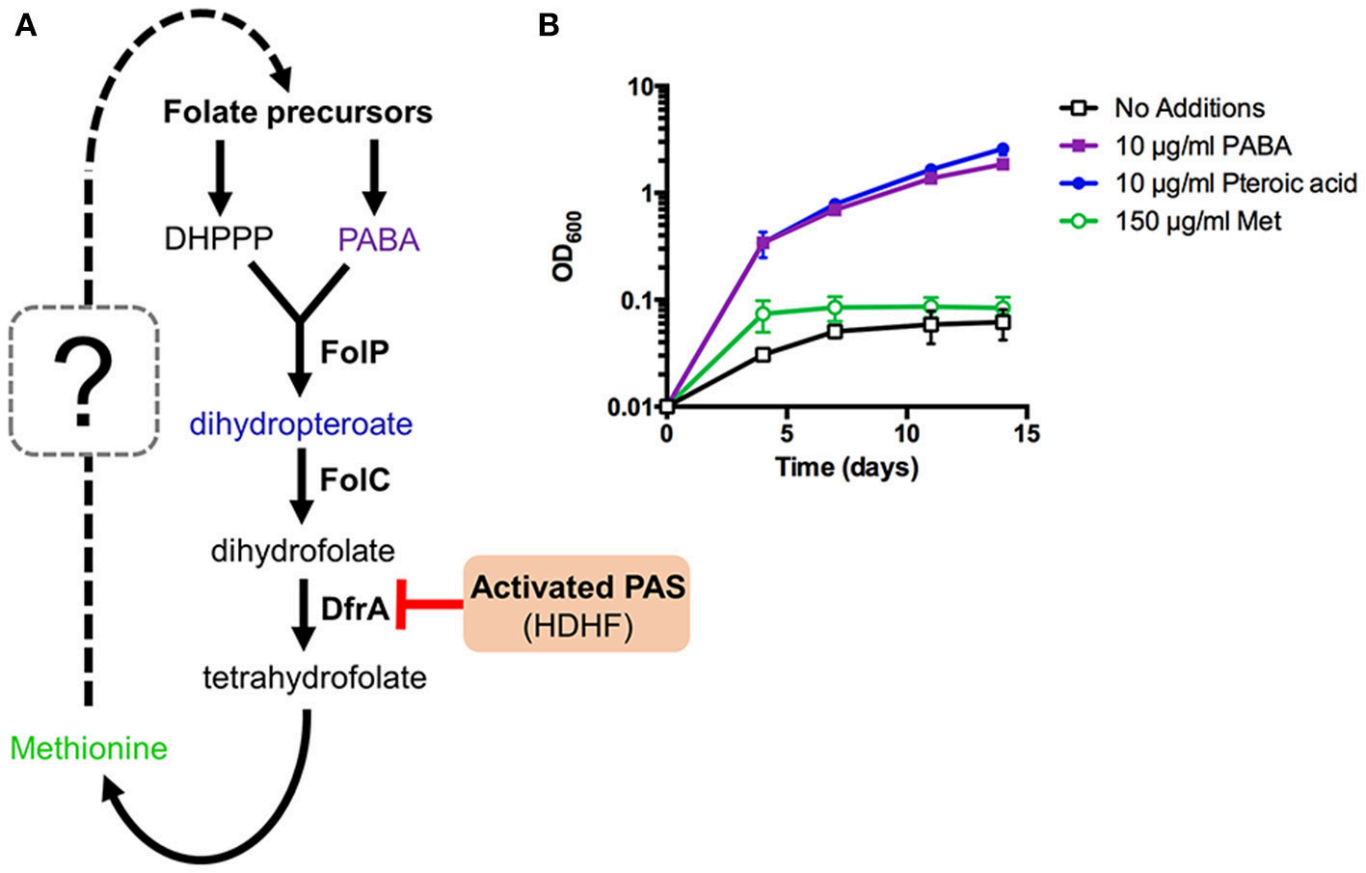
Methionine can affect but not bypass essentiality of upstream folate biosynthetic pathways in *M. tuberculosis*. **(A)** New working model of methionine-mediated PAS antagonism. **(B)** *M. tuberculosis* Δ*pabB* was grown to an OD_600_ of ~0.5, washed three times with PABA-free 7H9 medium to remove residual PABA and resuspended in PABA-free 7H9 medium to a starting OD_600_ of 0.01. Cultures were then supplemented with the indicated metabolites and incubated for 14 days with OD_600_ readings taken at the given time points.

It is also known that intracellular PABA levels affect PAS susceptibility in *M. tuberculosis* (Thiede et al., 2016). Since PABA biosynthesis is essential for mycobacterial growth, we hypothesized that PABA biosynthesis could be associated with methionine-mediated antagonism of PAS. PabB, aminodeoxychorismate synthase, is one of the essential enzymes required to convert chorismate to PABA in *M. tuberculosis* (Figure 4A). Consequently, a *M. tuberculosis* H37Ra *pabB* deletion strain is a PABA auxotroph and relies upon exogenous sources of PABA for growth (Figure 4B). The folate precursor dihydropteroate is produced from PABA and DHPPP (Figure 4A). We found that pteroic acid, an oxidized form of dihydropteroate can also support the growth of the *M. tuberculosis* H37Ra *pabB* deletion strain (Figure 4B). As expected, unlike PABA and pteroic acid, methionine did not support the growth of the *M. tuberculosis* H37Ra *pabB* deletion strain indicating that methionine alone is insufficient to fulfill cellular folate requirements in PABA starved *M. tuberculosis* cells. Using the *M. tuberculosis* H37Ra Δ*pabB* strain, we tested the requirement of PABA biosynthesis on methionine-mediated PAS antagonism. We observed that methionine potently antagonized PAS susceptibility in wild type *M. tuberculosis* H37Ra. In contrast, PAS susceptibility of the *M. tuberculosis* H37Ra Δ*pabB* strain was not antagonized by the addition of methionine (Table 3). Taken together, these data demonstrated that a functional PABA biosynthetic pathway is essential for methionine to antagonize PAS in *M. tuberculosis*.

### Biotin cofactor biosynthesis is essential for intrinsic resistance to PAS and other anti-tubercular drugs

**Figure 5:**
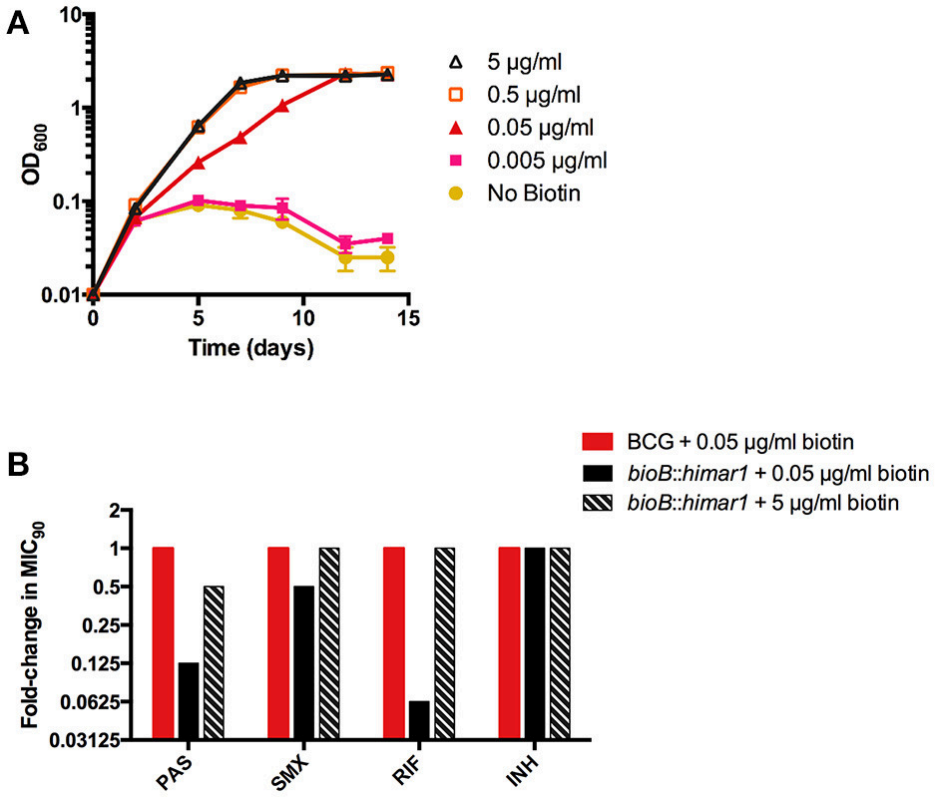
Disruption of *bioB* is growth inhibitory and potentiates drug action. **(A,B)** *M. bovis* BCG *bioB*::*himar1* was grown to an OD_600_ of ~0.5, washed three times to remove residual biotin with biotin-free 7H9 medium, and resuspended in biotin-free 7H9 medium to a starting OD_600_ of 0.01. Cultures were then supplemented with biotin and incubated for 14 days with OD_600_ readings taken at the given time points. Error bars denote standard deviation and are representative of 2 separate experiments. **(B)** *M. bovis* BCG and *bioB*::*himar1* were grown to an OD_600_ of ~0.5, washed three times to remove residual biotin, and resuspended in biotin-free 7H9 medium to a starting OD_600_ of 0.01. Cultures were then supplemented with biotin (0.05 and 5) and incubated for 14 days with OD_600_ readings taken at the given time points. MIC_90_ is defined as the minimum concentration of inhibitor required to restrict at least 90% of growth relative to growth seen in the no-drug control cultures. PAS, *para*-aminosalicylic acid; SMX, sulfamethoxazole; RIF, rifampin; INH, isoniazid. Results shown are representative of 3 separate experiments.

Our screening also identified several mutations that conferred increased susceptibility to PAS even in the absence of methionine. One strain, harboring a *himar1* insertion within *bioB*, encoding biotin synthase, showed increased susceptibility to PAS both in the presence and absence of methionine (Table 2). BioB is a radical SAM-dependent enzyme required for the final step in the synthesis of biotin. We confirmed the *bioB*::*himar1* strain exhibited biotin auxotrophy (Figure 5A). Chemical complementation of biotin revealed that 0.05 μg/ml biotin supplementation was barely sufficient to support near wild-type level growth. We speculated that susceptibility of the *bioB*::*himar1* strain to PAS was dependent upon the intracellular concentration of biotin. Thus, we examined the PAS susceptibility of the *bioB*::*himar1* strain using media containing minimal (0.05 μg/ml) or excess (5 μg/ml) concentrations of biotin (Figure 5B). We observed the *bioB*::*himar1* strain was far more susceptible to PAS (8-fold decrease in MIC_90_) in minimal biotin medium. In contrast, PAS susceptibility of the *bioB*::*himar1* strain was restored back to near wild-type level in excess biotin medium. Interestingly, the *bioB*::*himar1* strain was also more susceptible to sulfamethoxazole and rifampicin in minimal biotin medium, but maintained wild-type susceptibility to isoniazid, indicating that alterations in susceptibility profiles are drug-specific (Figure 5B). Similar to PAS, sulfamethoxazole and rifampicin susceptibilities of the *bioB*::*himar1* strain were restored to wild-type level in the excess biotin medium. Since PAS, sulfamethoxazole, and rifampicin susceptibilities of the *bioB*::*himar1* were dependent upon the concentration of supplemented biotin, these results indicated that defect in biotin cofactor biosynthesis in the *bioB*::*himar1* strain is responsible for increased susceptibility to PAS, sulfamethoxazole and rifampicin.

## Discussion

Methionine is the only folate-dependent metabolite known to antagonize certain anti-folate drugs in *M. tuberculosis* and other bacterial species. Interestingly, anti-folate drugs antagonized by methionine are also antagonized by PABA, a folate precursor. Although the molecular mechanism of PABA-mediated anti-folate antagonism is well-understood, how methionine antagonizes anti-folate drugs has yet to be elucidated. Our findings revealed that methionine-mediated PAS antagonism is linked to synthesis of folate precursors.

One strain isolated in our screen harboring a *himar1* disruption within the amino acid permease MetM fully sensitized *M. bovis* BCG to PAS in the presence of normally antagonistic concentrations of methionine and could be rescued by complementation of the *metM* disruption. In addition, the *himar1* disruption within *metM* prevented *M. bovis* BCG from assimilating sulfur derived from methionine and significantly decreased uptake of L-methionine-(*methyl*-d_3_). MetM belongs to the APC superfamily of transporters and our data suggested that MetM is principally responsible for uptake of methionine *in vitro*. Our data also suggest the existence of a secondary transporter for methionine based upon the decreased, but still detectable, accumulation of L-methionine-(*methyl*-d_3_) in *M. bovis* BCG *metM*::*himar1*. The most well-studied methionine transport system in bacteria is the MetD ABC transporter system of the methionine uptake transporter family found in numerous organisms including *E. coli* and even the closely related non-tubercular Mycobacterium, *Mycobacterium abscessus* (Gál et al., 2002). In *E. coli*, the MetD ABC transporter is encoded by the *metNIQ* gene cluster (Merlin et al., 2002). The *M. tuberculosis* complex has no known orthologs of this system, despite the known bioavailability of methionine in human and mouse serum (Lewis et al., 1980; Rivera et al., 1987). To our knowledge, this study represents the first characterization of a methionine transporter in the *M. tuberculosis* complex. Orthologs of MetM with high amino acid sequence similarities are found from *Gordonia sputi, Bacillus subtilis* and *Lactococcus lactis* and an ortholog from *L. lactis* has been shown to transport branched-chain amino acids, along with methionine (den Hengst et al., 2006). Existence of a conserved methionine transporter within the mycobacterium complex would be intriguing given that methionine/SAM biosynthesis is indispensable for survival of *M. tuberculosis* in murine and macrophage models of infection (Berney et al., 2015).

We also found that methionine-mediated PAS antagonism does not appear to occur through *N*,*N*-dimethylation by SAM-dependent methyltransferase(s). We addressed this possibility because *N*,*N*-dimethyl PAS, an inactive metabolite of PAS, was previously identified in metabolite extracts from PAS treated *M. tuberculosis* (Chakraborty et al., 2013). In addition, a SAM-dependent methyltransferase (*Rv0560c*) is induced by salicylate and salicylate analogs, including PAS (Schuessler and Parish, 2012). However, a recent report described that an unmarked in-frame deletion of *Rv0560c* in *M. tuberculosis* conferred no alteration in susceptibility to PAS, or other antimicrobials *in vitro* (Kokoczka et al., 2017). Consistent with this finding, our screen did not identify *Rv0560c*::*himar1* mutants. Together with our observation that pterin-PAS is also antagonized by methionine, we conclud that methionine-mediated PAS antagonism is not likely via PAS inactivation by *N*,*N*-dimethylation.

Importantly, methionine was unable to antagonize PAS in a *pabB* deletion mutant strain indicating that methionine-mediated PAS antagonism is dependent upon a functional PABA biosynthetic pathway. This finding is consistent with past and recent reports that methionine only antagonizes the anti-folate drugs that are also antagonized by PABA (Huang et al., 1997; Zheng et al., 2013; Nixon et al., 2014; Zhao et al., 2016). While the metabolic connections linking methionine to folate precursor biosynthesis remain to be determined, the DHPPP pathway has been shown to modulate susceptibility of *E. coli, Salmonella enterica* and *Burkholderia pseudomallei* to sulfamethoxazole (Li et al., 2017; Podnecky et al., 2017; Minato et al., 2018), which is predicted to be metabolically linked with methionine-mediated antagonism (Minato and Baughn, 2017). Further, we recently demonstrated that the biosynthetic pathway to DHPPP is essential for methionine-mediated antagonism of sulfonamide action in *E. coli* (Minato et al., 2018).

One PAS-sensitive mutant strain with a disruption in biotin synthase (*bioB*) was found to be auxotrophic for the cofactor biotin. Characterization of this mutant confirmed that disruption of biotin biosynthesis could enhance susceptibility to PAS and rifampicin in biotin-limited conditions. In *M. tuberculosis*, biotin is a cofactor required for acyl-CoA-carboxylase (ACC) enzymes participating in key metabolic processes in lipid biosynthesis (Takayama et al., 2005; Gago et al., 2011; Salaemae et al., 2011; Woong Park et al., 2011). Biotin biosynthesis and protein biotinylation process have been targeted for novel drug development (Duckworth et al., 2011; Shi et al., 2013; Tiwari et al., 2018). On the basis of our *in vitro* findings, targeting biotin synthesis may promote accumulation of antimycobacterial drugs by disrupting cell envelope integrity, which could revitalize drug therapies that are unable to overcome the relatively impermeable cell envelope of *M. tuberculosis* at clinically relevant dosages. Indeed, it was recently reported that disruption of protein biotinylation potentiates rifampicin activity against *M. tuberculosis* (Tiwari et al., 2018). It was previously reported that biotin has a vital role in methionine-mediated, PAS antagonism, such that supplementation with exogenous biotin was required to observe antagonism by methionine (Hedgecock, 1956). However, our study found that biotin supplementation was non-essential for methionine to antagonize PAS in *M. bovis* BCG suggesting that the effect of biotin on PAS susceptibility is independent of the precise mechanism of antagonism, and the initial observations in *M. tuberculosis* by Hedgecock may be a strain specific phenotype.

In summary, the mechanistic basis of methionine-mediated PAS antagonism was examined. Over 30 novel modulators of PAS susceptibility were identified by *himar1* transposon mutagenesis. However, with exception of the methionine transporter MetM, none of the functions identified were found to be directly involved in antagonism. Upon closer examination, *de novo* biosynthesis of PABA was determined as essential for methionine-mediated antagonism, revealing a previously unappreciated relationship between methionine and folate precursor synthesis. Further studies are needed to reveal the precise mechanism of this process. The results presented here also identified tractable drug targets within *M. tuberculosis* that could be exploited to enhance antimycobacterial drug action.

## Author contributions

MH, SK, MC, and AAB performed experiments. ADB, CA, and YM conceived the work. MH, ADB, and YM wrote the manuscript. All authors contributed to analysing data and editing of the manuscript.

### Conflict of interest statement

The authors declare that the research was conducted in the absence of any commercial or financial relationships that could be construed as a potential conflict of interest.
