# Supplementary material for: Methionine Antagonizes para-Aminosalicylic Acid Activity via Affecting Folate Precursor Biosynthesis in Mycobacterium tuberculosis
Source: Front Cell Infect Microbiol. 2018 Nov 12;8:399. doi: 10.3389/fcimb.2018.00399 (PMC6240602; doi:10.3389/fcimb.2018.00399)
Supplement: Supplementary file 1 [file Table_1.DOCX]

Supplementary Material

**Methionine antagonizes *para*-aminosalicylic acid activity via affecting folate precursor biosynthesis pathway in *Mycobacterium tuberculosis***

Michael D. Howe^1^, Shannon L. Kordus^1^, Malcolm S. Cole^2^, Allison A. Bauman^1^, Courtney C. Aldrich^2^, Anthony D. Baughn^1*^, Yusuke Minato^1*^

^1^Department of Microbiology and Immunology, University of Minnesota Medical School, Minneapolis, MN, USA.

^2^Department of Medicinal Chemistry, University of Minnesota, Minneapolis, MN, USA

*** Correspondence:** Correspondence and requests for materials should be addressed to YM (email: yminato@umn.edu) or ADB (email: [abaughn@umn.edu](mailto:abaughn@umn.edu))

**Supplementary Table S1. List of primers used in this study**

| **Name** | **Sequence (5’-3’)** | **Source** | **Restriction Site** |
| --- | --- | --- | --- |
| BCG3282c_For | TTTTTTGCTAGCTCAACACCTCCGGGTCG | This work | *Nhe*I |
| BCG3282c_Rev | TTTTTTGAATTCATGGCCGGTCGACGGCGCATG | This work | *Eco*RI |
| p0004s_Hygro+P_F | TTTTCTTAAGTGTTGTCAAAGACAGCATCCT | (Thiede et al. 2016) | *Afl*II |
| p0004s_Hygro+P_R | TTCCTGCAGGTCAGGCGCCGGGGGCGG | (Thiede et al. 2016) | *Sbf*I |
| pTIC6a_F | TTTCCTGCAGGGTAACACTGGCAGAGCATTACGC | (Thiede et al. 2016) | *Sbf*I |
| pTIC6a_R | TTTTCTTAAGGAGCAAGACGTTTCCCGTTGA | (Thiede et al. 2016) | *Afl*II |

**Supplementary Table S2. Mass spectrometry transitions and parameters**

| **Compound** | **Precursor Ion** | **Product Ion** | **Collision Energy (V)** |
| --- | --- | --- | --- |
| L-methionine-(*methyl*-d_3_) |  |  | **15** |
| N-phenyl glycine  (Internal standard) |  |  | **19** |

Declustering Potential (DP): 46.0 V, Entrance Potential (EP): 10.0 V, Collision Cell Exit Potential (CXP): 10.0 V were kept constant for all transitions.

**Supplementary Table S3**. Susceptibility of *M. bovis* BCG and *metM*::*himar1* to PAS and other first-line antimycobacterial drugs.

| **Drug** | ***M. bovis* BCG MIC_90_*^a^* (µg/ml)** | ***M. bovis* BCG *metM*::*himar1* MIC_90_ (µg/ml)** |
| --- | --- | --- |
| PAS | 1 | 1 |
| RIF | 0.06 | 0.06 |
| INH | 0.04 | 0.04 |

*^a^*MIC^90^ is defined as the minimum concentration of drug required to restrict at least 90% of growth relative to growth seen in the no-drug control cultures. PAS, *para*-Aminosalicylic acid; RIF, rifampin; INH, isoniazid.
